# Antemortem diagnosis of anomalous origin of the left coronary artery from the pulmonary artery in a dog

**DOI:** 10.1186/s12917-022-03165-z

**Published:** 2022-02-19

**Authors:** Kazuki Takamura, Ayaka Chen, Shin Ono, Masami Uechi

**Affiliations:** 1 JASMINE Veterinary Cardiovascular Medical Center, 1-8-37 Nakagawa, Tsuzuki, Yokohama, Kanagawa 224-0001 Japan; 2 SKYVETS, Inc., 2958-4 Yanokuchi, Inagi, Tokyo, 206-0812 Japan

**Keywords:** Canine, Congenital heart disease, Coronary artery anomaly

## Abstract

**Background:**

In both humans and animals, anomalous origin of the left coronary artery from the pulmonary artery (ALCAPA) is a rare congenital coronary artery anomaly. In veterinary medicine, ALCAPA is reported to be discovered only during autopsy or necropsy, and diagnostic methods and prognosis remain poorly understood in dogs.

**Case presentation:**

A 6-month-old Kaninchen Dachshund was diagnosed with functional mitral valve regurgitation and ALCAPA. Echocardiography identified anomalous vessels in the left ventricular wall and abnormal origin of the left coronary artery from the pulmonary artery.

Further evaluation with coronary computed tomographic angiography demonstrated the left coronary artery arising from the posterior aspect of the main pulmonary artery together with the characteristic findings of ALCAPA. The right coronary artery was found to be dilated and tortuous. Furthermore, dilated coronary collateral arteries within the ventricular septum and along the epicardial surface were observed. The dog underwent surgery, but the origin of the anomalous artery could not be ligated, and it died from pulmonary edema 5 months after surgery.

**Conclusion:**

Anomalous origin of the left coronary artery from the pulmonary artery is overlooked in clinical practice due to its rarity. Coronary computed tomographic angiography was useful to definitively diagnose ALCAPA in a low-invasive manner. Antemortem diagnosis of ALCAPA was shown to be possible in dogs for the first time, and presence of unexplained mitral valve regurgitation should raise concern to this anomaly.

## Background

Anomalous left coronary artery from the pulmonary artery (ALCAPA) is a rare congenital heart disease in humans that usually presents as an isolated heart defect [1–3]. As its name suggests, the left coronary artery (LCA) arising from the main pulmonary artery (PA) is the diagnostic hallmark of the disease. Of the two types of ALCAPA, infantile or adult, the adult variant is much rarer, as this malformation induces death in the initial stage of life if left untreated [3, 4]. Until the neonatal phase, ALCAPA does not interfere with the hemodynamics of the coronary arteries, and no difference in blood pressure occurs between the systemic and pulmonary arterial circulation through the presence of ductus arteriosus. When the ductus arteriosus closes after birth, pulmonary arterial circulation pressure decreases physiologically, consequently lowering the blood flow rate through the LCA [1, 3].

In veterinary medicine, ALCAPA is also a rare disorder discovered during autopsy or necropsy [5–8], and valid diagnostic methods or prognosis remain unclear in dogs. This report demonstrates the validity of coronary computed tomography angiography (CCTA) aided with echocardiography in diagnosing ALCAPA in a small breed dog. This will support clinicians in diagnosing the disease while the dogs are alive and in making proposals for treatments.

## Case presentation

A 6-month-old female Kaninchen Dachshund with a tentative diagnosis of mitral valve dysplasia was referred to our center for further evaluation. The dog was smaller than its littermates and had initially presented to a veterinarian at 4 months of age due to a history of tachypnea. A cardiac murmur was auscultated, and echocardiography revealed severe mitral valve regurgitation (MR) with left ventricular and atrial enlargement. Pimobendan^1^ (0.58 mg/kg PO q12h), alacepril^2^ (3.9 mg/kg PO q12h), torasemide^3^ (0.1 mg/kg PO q12h), and isosorbide dinitrate^4^ (2.0 mg/kg PO q12h) were prescribed as treatment for MR and pulmonary edema. The patient was referred to the university animal hospital for further evaluation, where mitral dysplasia with abnormal coronary vessels was diagnosed.

On presentation to JASMINE, the patient was lean with a body condition score of 4/9 and weighed 3.36 kg. A grade 3/6 left apical systolic murmur was auscultated. The femoral pulses were normal and synchronous, and lung sounds were normal in all areas. The patient was alert with a heart rate of 140 bpm, a respiratory rate of 48 breaths/min, and a temperature of 38.9 °C. Complete blood count, plasma biochemistry, and cardiac biomarker evaluation were conducted. Abnormalities detected included elevated NT-proBNP (4887 pmol/L; reference range ≤ 900 pmol/L) and ANP (259.9 pg/mL; reference range 8.6–105.8 pg/mL). Cardiac troponin I was normal (0.016 ng/mL; reference range 0.006–0.129 pg/mL).

**Fig. 1:**
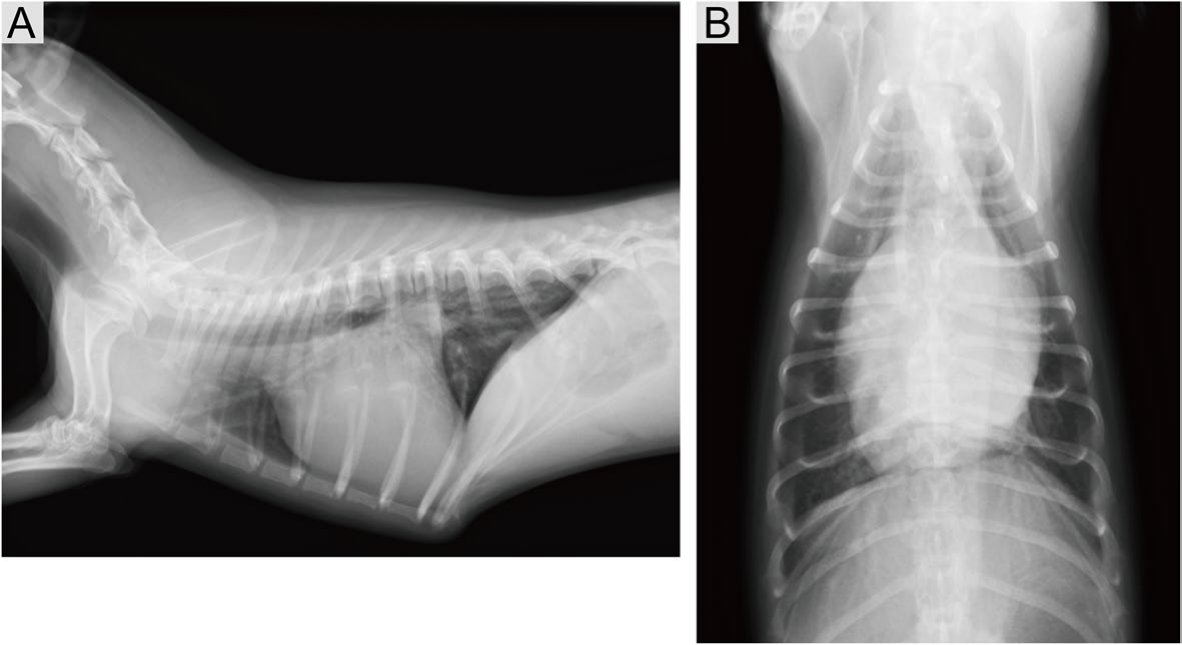
**A** Right lateral and **B** dorsoventral thoracic radiograph demonstrating severe cardiomegaly. The pulmonary parenchyma appears normal

Systemic blood pressure (oscillometry) was 118/50/77 mmHg (systolic/diastolic/mean). A six-lead electrocardiography performed in the right lateral recumbency demonstrated sinus rhythm with one criterion for left ventricular enlargement (R wave amplitude of 3.1 mV in lead II). However, occasional premature ventricular contraction was observed on monitor electrocardiography during transthoracic echocardiography (TTE). The right lateral and dorsoventral thoracic radiographs (Fig. 1A and B) revealed severe cardiomegaly (vertebral heart size, 11.6 v) [9] and left atrial (LA) enlargement (vertebral left atrium size, 2.8 v) [10], with no evidence of pulmonary edema.

**Fig. 2:**
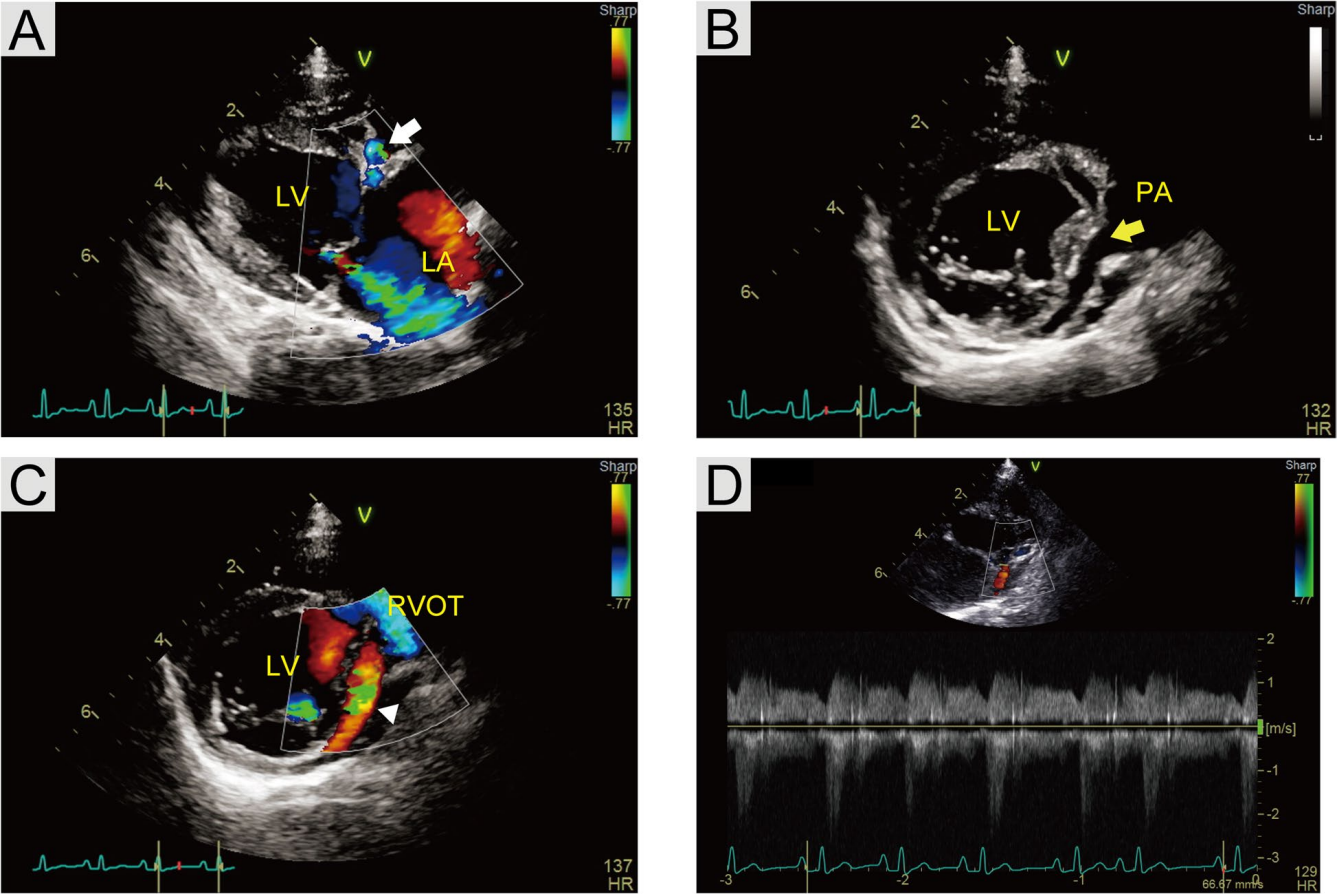
**A** Color Doppler image of right parasternal long-axis view demonstrating moderate mitral valve regurgitation and color flow through coronary collaterals in the interventricular septum (white arrow). **B** Right parasternal short-axis view demonstrating the anomalous origin of the left coronary artery from the pulmonary artery (yellow arrow). **C** Color Doppler image of right parasternal short-axis view at the mitral valve level, demonstrating the retrograde flow in the LCA to the PA (white arrowhead). **D** Continuous wave Doppler flow tracing demonstrating continuous, mostly systolic retrograde flow, in the dilated coronary artery. LA: left atrium; LV: left ventricle; PA: pulmonary artery; RVOT: right ventricular outflow tract

The transthoracic echocardiogram revealed severe LA dilation (2D short axis left atrial to aortic ratio 2.12) [11]. The left ventricle (LV) was enlarged (M-mode left ventricular internal dimension in end-diastole normalized to bodyweight 2.33) [11] with mildly reduced systolic function (M-mode left ventricular internal dimension in end-systole normalized to bodyweight 1.55 [12] and fractional shortening 31.5% [12]). However, no apparent abnormalities were observed in LV wall movement, subjectively. Color Doppler analysis was obtained through a right parasternal long-axis four-chamber view, and the mitral valve showed a moderate regurgitation signal (Fig. 2A). No structural abnormalities were found in the mitral valve complex, which consists of the mitral valve, mitral valve annulus, and papillary muscles; however, the leaflet coaptation area was decreased. The blood flow of multiple aberrant vessels was observed within the inter-ventricular septum in the right parasternal long-axis four-chamber view (Fig. 2A). Large aberrant vessels were most prominent within the LV wall and were best visualized with color Doppler (Fig. 2C). The continuous wave Doppler analysis of the aberrant vessels revealed continuous systodiastolic flow with a maximal velocity diastole (velocity, 1.20 m/s; pressure gradient, 5.76 mmHg) and a minimal velocity systole (velocity, 0.86 m/s; pressure gradient, 2.94 mmHg) (Fig. 2D). The left coronary artery (LCA) optimum was not detected in the left coronary sinus, and the trunk of the coronary artery was directly connected to the PA (Fig. 2B). After these examinations, torasemide^3^ dosage was increased from 0.1 mg/kg PO q12h to 0.2 mg/kg PO q12h in order to reduce the volume overload.

**Fig. 3:**
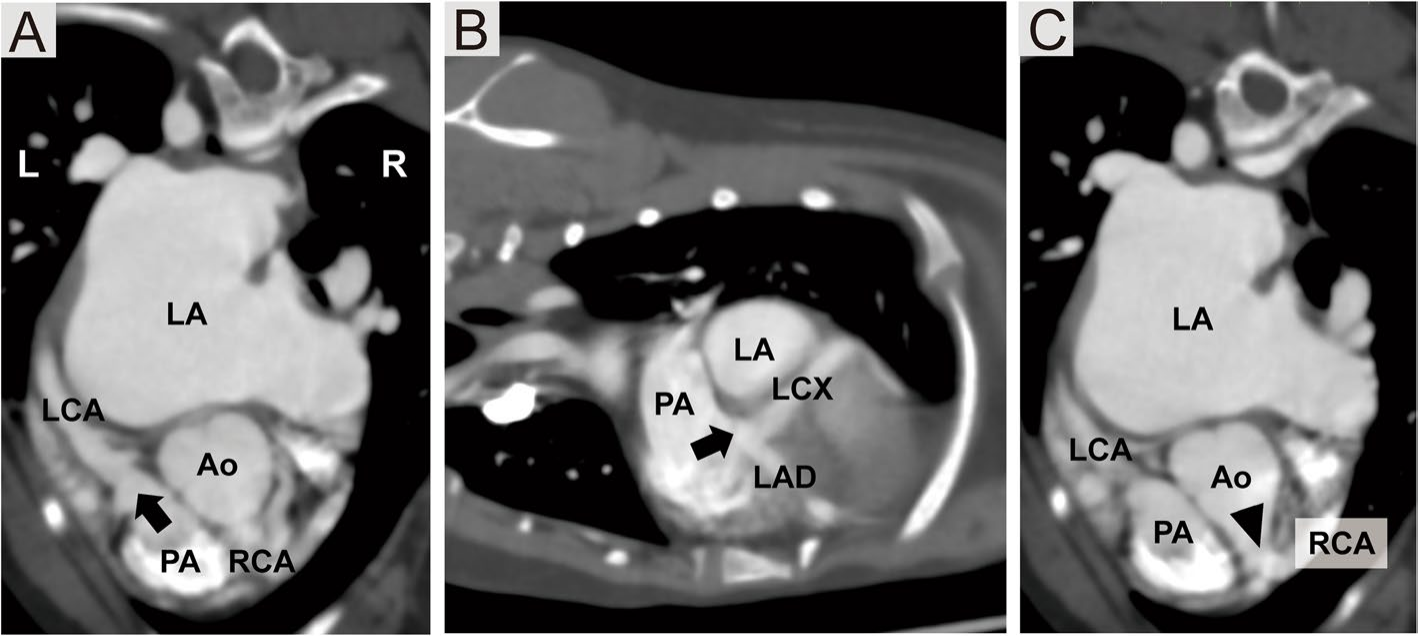
**A** Axial and **B** sagittal coronary computed tomographic angiographic images demonstrating the origin of the left coronary artery (arrow) from the pulmonary artery. **C** Axial coronary computed tomographic angiographic images demonstrating the origin of the right coronary artery (arrowhead) from the aorta. The entire right coronary artery appears enlarged. Ao: aorta; LAD: left anterior descending artery; LCA: left coronary artery; LCX: left circumflex artery; PA: pulmonary artery; RCA: right coronary artery

**Fig. 4:**
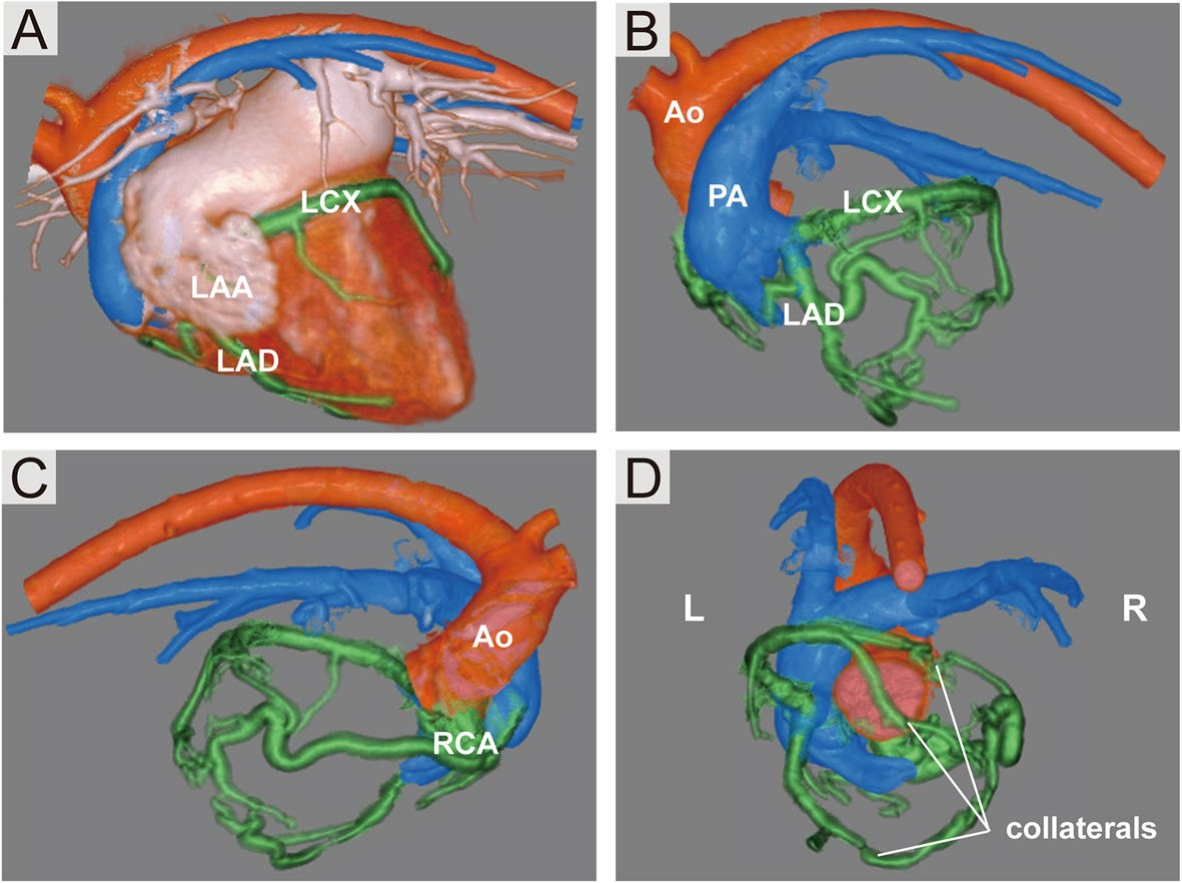
Three-dimensional volume-rendered computed tomography angiography images. **A**, **B** The left coronary artery anomaly originates from the pulmonary artery. **C** The right coronary artery is tortuous and dilated. **D** Prominent collateral vessels between RCA, LCX, and LAD. Ao: aorta; LAA: left atrial appendage; LAD: left anterior descending artery; LCX: left circumflex artery; PA: main pulmonary artery; RCA: right coronary artery

An ECG-gated CCTA of the thorax was performed under general anesthesia using a multidetector 80-row computed tomography scanner^5^ in the helical mode to obtain a detailed anatomic image of the abnormal vessels. The scanner was positioned to encompass the ascending aorta. Images were acquired at 10 and 90% of the R–R interval. Acquisition parameters of the scanning were as follows; spiral pitch factor: 0.175, tube voltage: 120 kVp, reference tube current: 250 mV, slice thickness: 0.5 mm, and a low frequency reconstruction algorithm. Contrast medium of 600 mg iodine/kg (2 mL/kg) was delivered via a power injector system^6^ into the cephalic vein at a rate of 0.7 mL/s to obtain an enhanced levophase. The examination revealed LCA and RCA originating from the PA and aorta, respectively (Fig. 3A, B and C, 4B and C). The dilation of RCA, LCA, and their branches; the left circumflex artery; and the left anterior descending artery (Fig. 4A, B and C) were also noted. The anomalous origin of the main LCA from the PA, and prominence of the right coronary circulation with an extensive network of collaterals that communicate and supply the LCA territory were found (Fig. 4D).

At the owner’s request, surgical interventions were performed 78 days after the first visit. The initial surgical plan was to use the internal thoracic artery as a bypass graft, but it was not sufficiently long to reach the left anterior descending artery (LAD). An alternative plan was applied using the left carotid artery to supply arterial blood to the LAD; the left carotid artery was dissected from the neck to the thoracic cavity and anastomosed to LAD under cardiopulmonary bypass. As the origin of LCA at the PA could not be confirmed and ligated, retrograde blood flow via LCA was recognized in the PA on post-operative echocardiography. Tachypnea resolved, but the same cardiac medications as prior to surgery was needed throughout the period after discharge since cardiomegaly did not improve. On 3 months follow-up, continuous systodiastolic flow was still observed in the aberrant vessels with no prominent changes in velocity compared to the preoperative echocardiographic values. The dog died from pulmonary edema 5 months after the surgery. Necropsy could not be performed due to lack of consent from the owner.

## Discussion and conclusions

This paper presents a very rare finding of MR which the principal cause lies in the coronary artery anomaly in a dog. To the author’s knowledge, this is the first report of an antemortem diagnosis of ALCAPA in a dog.

Echocardiography enables visualization of the abnormal origin of the LCA in humans [3, 13]. Dilated RCA, retrograde blood flow in the anomalous coronary artery, diastolic flow in the PA, and abnormal septal or epicardial color flow signals from the collateral vessels are common findings. Left ventricular dilation, left ventricular or global hypokinesis, and mitral insufficiency can also be identified. The adult variant of ALCAPA is characterized by compensatory collaterals between the RCA and LCA [4, 14, 15]. The echocardiographic findings in the presented case were similar to those of the human adult variant of ALCAPA and the collateral vessels explained the dog’s survival until 6 months of age. However, the RCA could not be visualized and none of the findings strongly support left ventricular hypokinesis. Of note, increased fractional shortening is generally observed under moderate to severe MR and volume overload, but as it was not observed in this case, a decrease in contractility was suggested.

For diagnosing coronary artery anomalies in human medicine, the performance of CCTA has proven to be highly viable [4, 15], for example, allowing visualization of the LCA arising from the main PA in ALCAPA. In the presented case, dilation of both the LCA and RCA with the presence of prominent collateral vessels was observed by CCTA, comparable with that in the human adult variant of ALCAPA. The origin of the LCA was located at the left inferolateral aspect of the main PA close to the pulmonary valve, which is representative of the human adult variant. Retrograde blood flow from the LCA to the PA was clearly observed that resembled a “steal phenomenon” described as the stealing of blood supply from the left coronary circulation provided by collateral vessels from the dilated right coronary system. The degree of collateral formation relates to the degree of successive myocardial ischemia [15], so it was hypothesized that the collateral vessels maintained oxygenated blood flow to the LCA territory for the dog in this case. The size of the dog in this case was smaller compared to recent reports using CCTA for visualization of coronary arteries [16, 17], but it was possible to clearly visualize the coronary artery. This case report supports the utility of CCTA as a diagnostic method for ALCAPA in dogs, as it depicts collaterals that cannot be clearly recognized by echocardiography alone.

As ALCAPA is an anatomical anomaly, surgery is required for definitive treatment [1–3, 15, 18, 19]. Operative strategies such as coronary button transfer (or coronary arterial translocation), Takeuchi repair, and use of a bypass graft with anomalous artery ligation have been established. In the case presented, since the distance between the anomalous coronary artery and aortic root was long, a coronary artery bypass graft of the left carotid artery and LAD was performed with cardiopulmonary bypass. However, the proximal anomalous artery was not ligated. Consequently, it is possible that the steal phenomenon was accelerated, with increased pulmonary blood flow, causing a further volume overload on the left heart. Therefore, in similar cases, ligation of the proximal anomalous artery is strongly recommended. Persistent cardiac enlargement observed during the period after surgery was explainable since the ligation was not achieved in the presented case. However, even if the dog had not taken surgery, it would eventually have faced the risk of death from cardiac failure as described in one dog with ALCAPA [5]. Improvement in tachypnea after surgery is likely the result of arterial blood supply to the LAD and there remains a possibility that the dog would have survived if ligation of the anomalous artery had succeeded.

To conclude, this study reports the first canine case of antemortem ALCAPA diagnosis, which can help veterinarians to arrive at this diagnosis in living dogs. CCTA is a low-invasive and common method to visualize coronary artery anomaly in humans and has proven to be applicable in small breed dogs. In young dogs showing cardiomegaly with mitral insufficiency, ALCAPA should be considered as a differential diagnosis, and a combination of TTE and CCTA is recommended for definitive antemortem diagnosis.

## Data Availability

Data sharing is not applicable to this article as no datasets were generated or analyzed during the current study.

## Abbreviations

ALCAPA: Anomalous origin of the left coronary artery from the pulmonary artery
CCTA: Coronary computed tomography angiography
LA: Left atrial
LAD: Left anterior descending artery
LCA: Left coronary artery
LV: Left ventricle
MR: Mitral valve regurgitation
PA: Pulmonary artery
RCA: Right coronary artery
TTE: Transthoracic echocardiogram

## Footnotes

1 Vetmedin® chewable 1.25 mg, Boehringer Ingelheim Animal Health Japan Co., Ltd., Tokyo, Japan

2 Apinac® 25 mg, DS Pharma Animal Health Co., Ltd., Osaka, Japan

3 Luprac® 4 mg, Mitsubishi Tanabe Pharma Corporation, Osaka, Japan

4 Nitrol® 5 mg, Eisai Co., Ltd., Tokyo, Japan

5 Aquilion PRIME, Canon Medical Systems Corporation, Tochigi, Japan

6 Oypalomin® 300 injection, Fuji Pharma Co., Ltd., Tokyo, Japan
